# Pump-free flushing coagulation: manual saline injection through the knife tip during gastric endoscopic submucosal dissection

**DOI:** 10.1055/a-2991-2135

**Published:** 2026-11-03

**Authors:** Hiroyuki Nakanishi, Shun Kuroiwa, Ryohei Kobayashi, Yutaka Yasui, Nobuharu Tamaki, Kaoru Tsuchiya, Masayuki Kurosaki

**Affiliations:** 1 Gastroenterology and Hepatology 26830 Musashino Red Cross HospitalMusashinoTokyoJapan; 2 Gastroenterology and Hepatology 26830 Musashino Red Cross HospitalTokyoJapan

Haemostasis during endoscopic submucosal dissection is difficult under immersion. The flushing method ejects saline from the knife base during activation, using a pump synchronised with the generator, removing the vaporisation layer and suppressing discharge. 1​ ​2​ It needs this pump, a specific knife and immersion; in the stomach, water-jet haemostasis has so far required a pressure pump. 3​

We describe a pump-free variant in which saline is injected from a syringe through a tip-type water-jet knife (ORISE ProKnife; Boston Scientific, USA), which delivers fluid from the needle tip rather than the sheath, 4​ so that saline reaches the electrode; only the electrode–tissue interface needs to be wetted. The operator signals the assistant before each activation and the flush continues through incision and coagulation, so the electrode is never dry.

A 79-year-old woman had a 0-IIc cardia lesion, endoscopically 20×10 mm; biopsy showed signet-ring-cell carcinoma, cT1a without ulceration, an absolute indication in the JGES guideline. Under CO _2_ insufflation, 24 vessels were precoagulated with forced coagulation, effect 6.5 (VIO 3; Erbe, Tuebingen, Germany), and transected with ENDO CUT I (2-2-1; Video 1 ). Four minor bleeds occurred, all controlled with the knife; haemostatic forceps were never needed. The procedure time was 49 minutes, without excessive fluid pooling; en bloc resection was achieved uneventfully ( Fig. 1 ). Histology showed a 27×15 mm signet-ring-cell carcinoma, pT1a, UL0, ly0, v0, free margins (eCura C-2).

**Video 1** Pump-free flushing coagulation and ENDO CUT transection of four submucosal vessels during gastric endoscopic submucosal dissection at the cardia, each manoeuvre replayed in slow motion. URI: .


**Fig. 1:**
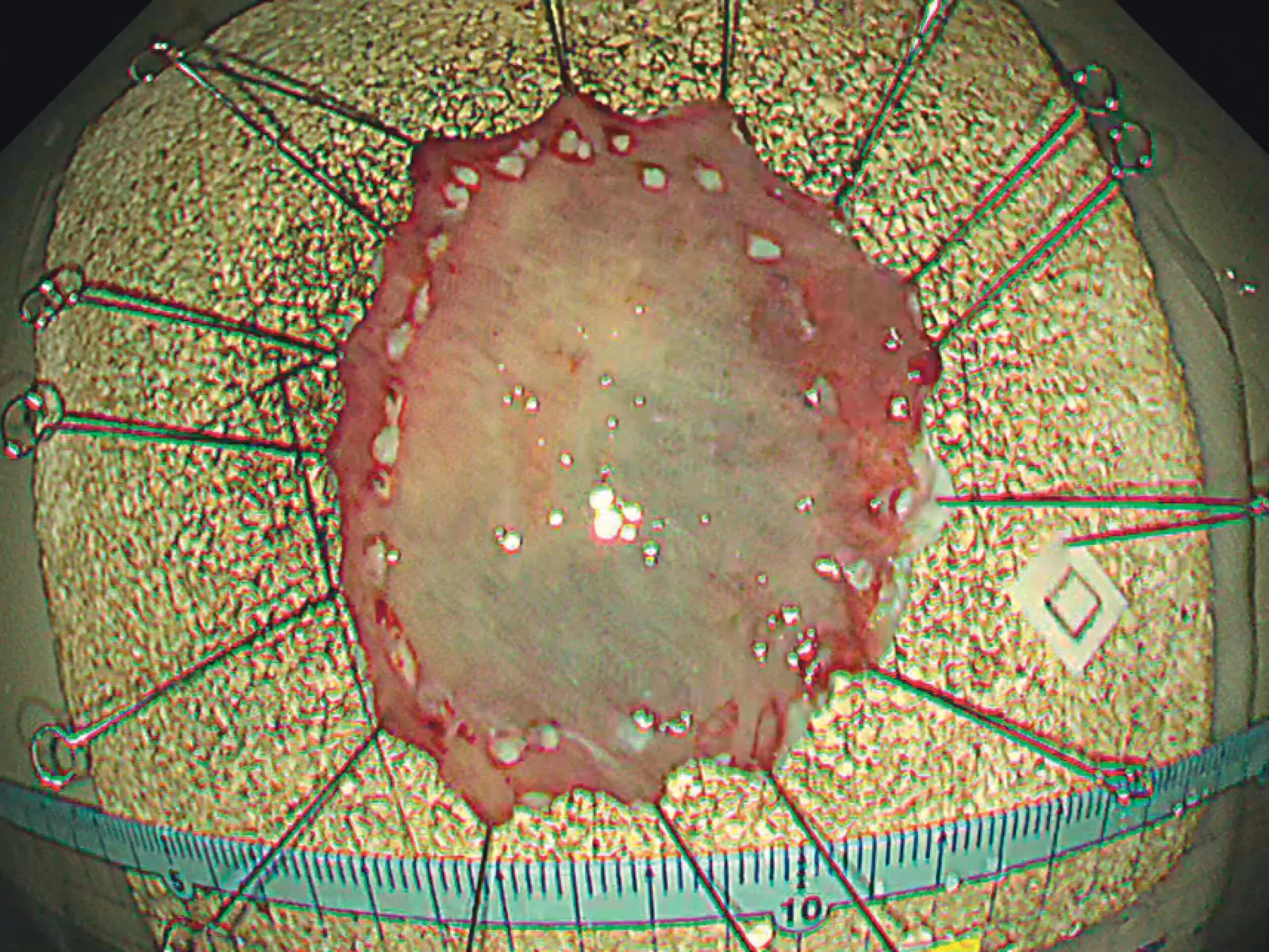
An en bloc resected specimen measuring 50×40 mm, pinned on a board alongside a centimetre scale.

Forced coagulation at effect 6.5 ordinarily sparks, scattering the current and risking bleeding and perforation. With the continuous flush, no spark occurred and the plane remained almost bloodless. The auxiliary water jet wets the mucosa, not the electrode, and cannot replace this. Delivering saline at the electrode tip may reproduce the flushing effect without a pump or immersion. This single case had no control; the technique depends on operator-assistant coordination and needs comparative study.

Endoscopy_UCTN_Code_TTT_1AO_2AG_3ADn

## Declaration of GenAI Use

A generative artificial intelligence large language model (Claude, Anthropic, San Francisco, California, USA) was used to assist with literature searching, with language editing of this manuscript and with technical processing of the video file. No clinical content, endoscopic images, video footage or data were generated or altered by the model. The authors reviewed and verified all the text, images and video, and take full responsibility for the content of this manuscript.
